# Serum Uric Acid and Creatinine Levels in Patients Receiving Anti-tuberculosis Therapy: A Cross-Sectional Study at a Tertiary-Care Hospital in Lahore, Pakistan

**DOI:** 10.7759/cureus.117659

**Published:** 2026-10-08

**Authors:** Kinza Parvez, Anum Rauf, Faiza Afzal, Ayesha Khan

**Affiliations:** 1 Chemical Pathology, King Edward Medical University, Lahore, PAK

**Keywords:** anti-tuberculosis therapy, hyperuricemia, pakistan, pyrazinamide, serum creatinine, serum uric acid, tuberculosis

## Abstract

Background: Anti-tuberculosis treatment is effective, but some drugs can cause biochemical abnormalities. Hyperuricemia is a recognized effect of pyrazinamide-containing therapy. Local information on serum uric acid and creatinine during routine anti-tuberculosis treatment is limited.

Objective: This study aimed to determine serum uric acid and creatinine levels and the frequency of hyperuricemia and elevated creatinine in patients receiving first-line anti-tuberculosis therapy and to assess associations between recorded treatment duration and these biochemical parameters, including an exploratory comparison between participants treated for ≤3 months and >3 months.

Methods: This cross-sectional laboratory-based study included 96 patients receiving first-line anti-tuberculosis therapy at Mayo Hospital, Lahore, from November 2025 to February 2026. Serum uric acid and creatinine were measured by the uricase-peroxidase and Jaffe kinetic alkaline picrate methods, respectively, on a Siemens Atellica automated chemistry analyzer. Continuous variables were summarized as mean ± standard deviation and median (interquartile range). As continuous variables departed from normality, Spearman rank correlation was used to assess associations between treatment duration and biochemical parameters. The Mann-Whitney U test was used for the exploratory comparison between duration groups, and Fisher's exact test was used for hyperuricemia.

Results: Mean age was 30.15 ± 12.75 years, and mean treatment duration was 4.38 ± 2.92 months. Fifty-four participants (56.3%) had pulmonary tuberculosis, and 42 (43.8%) had extrapulmonary tuberculosis. Median uric acid was 5.00 mg/dL (IQR 3.90-6.63; mean 5.62 ± 2.51), and median creatinine was 0.72 mg/dL (IQR 0.62-0.80; mean 0.71 ± 0.18). Hyperuricemia occurred in 19 (19.8%; 95% CI 13.1-28.9%) participants and elevated creatinine in 2 (2.1%; 95% CI 0.6-7.3%). Treatment duration showed a weak inverse correlation with uric acid (Spearman ρ = −0.213, p = 0.037), but not with creatinine (ρ = 0.066, p = 0.522). Uric acid was higher in the ≤3-month group than in the >3-month group (6.52 ± 3.14 vs. 4.82 ± 1.39 mg/dL; p = 0.005). Hyperuricemia was also more frequent in the ≤3-month group (35.6% vs. 5.9%; p < 0.001). Male participants had higher uric acid than female participants (6.46 ± 2.86 vs. 4.96 ± 1.99 mg/dL; p = 0.002). Neither parameter differed significantly by case status or tuberculosis site.

Conclusion: Hyperuricemia occurred in approximately one-fifth of participants receiving first-line anti-tuberculosis therapy, whereas elevated creatinine was uncommon. Although hyperuricemia was more frequent in the ≤3-month group, treatment regimen and pyrazinamide exposure were not independently verified against drug charts, and pretreatment biochemical values were unavailable. Therefore, the duration-defined difference cannot establish a treatment-related effect or specifically attribute hyperuricemia to pyrazinamide exposure. Prospective studies with serial measurements and verified drug exposure are needed.

## Introduction

Tuberculosis (TB) remains an important infectious disease worldwide and requires treatment with several drugs for a prolonged period. Pakistan is among the countries with the highest tuberculosis burden globally, accounting for a substantial share of new cases each year [1]. Current World Health Organization guidance includes different treatment regimens according to the type of tuberculosis and drug susceptibility. Common first-line drugs include isoniazid, rifampicin, pyrazinamide, and ethambutol. Although these medicines are effective, they can also produce biochemical changes during treatment [2,3].

Hyperuricemia is a well-known biochemical effect of pyrazinamide-containing treatment. Pyrazinamide and its metabolites reduce renal uric acid excretion, which can result in an increase in serum uric acid [4,5]. A Pakistani study involving 216 patients receiving pyrazinamide-containing therapy reported a marked rise in serum uric acid during the early weeks of treatment, followed by a decrease after pyrazinamide was stopped [6]. Another Pakistani cohort similarly reported a high frequency of hyperuricemia and accompanying arthralgia during pyrazinamide-containing treatment [7]. A longitudinal study also reported a clear increase in serum uric acid during the first two months of treatment, followed by a return toward baseline after the pyrazinamide-containing phase [8].

Serum creatinine is commonly used to assess renal function [9]. However, an increased creatinine value alone does not establish acute kidney injury or prove that anti-tuberculosis drugs are responsible for renal injury [9]. Acute kidney injury has been associated with increased mortality among patients with tuberculosis, which underscores the importance of monitoring renal function during treatment [10]. Renal function during treatment may also be affected by the severity of tuberculosis, other illnesses, concomitant medicines, and the treatment regimen itself.

Local data on the frequency of hyperuricemia and renal biochemical abnormalities during anti-tuberculosis therapy are limited. Evidence on their relationship with treatment duration and phase is also scarce in Pakistan. This study was therefore undertaken at Mayo Hospital, Lahore, to characterize these biochemical abnormalities in patients receiving first-line anti-tuberculosis therapy and to explore their relationship with treatment duration and phase.

## Materials and methods

Study design and setting

This was a cross-sectional, laboratory-based study conducted at Mayo Hospital, Lahore, Pakistan, from November 2025 to February 2026. Patients receiving anti-tuberculosis treatment were recruited from the TB ward and outpatient department. Serum uric acid and creatinine were analyzed in the Central Diagnostic Laboratory of Mayo Hospital. This study is reported in accordance with the STROBE (Strengthening the Reporting of Observational Studies in Epidemiology) statement for cross-sectional studies [11]. Because this was a cross-sectional study, each participant contributed a single blood sample corresponding to their treatment duration at the time of enrollment; pretreatment (baseline) serum uric acid and creatinine values were not available for comparison.

Sample size and sampling

The required sample size was calculated using the single-proportion formula:

$n = \frac{[Z_{(1-\alpha/2)}]^2 \times p \times q}{d^2}$

where n is the required sample size; Z is the standard normal deviate corresponding to the desired confidence level, read from the standard normal distribution for a two-tailed significance level of α (for a 95% confidence level, α = 0.05, so α/2 = 0.025 in each tail, giving Z_(1−α/2)_ = 1.96); p is the expected proportion of patients with altered serum uric acid levels (p = 0.60, based on prior local data [12]); q = 1 − p; and d is the absolute precision, or margin of error, allowed around this proportion (d = 0.10). Substituting these values gave a required sample size of 96 participants. Non-probability consecutive sampling was used. A total of 96 participants were recruited, and all 96 were retained in the final analysis.

Eligibility criteria

Patients aged 18-70 years with pulmonary or extrapulmonary tuberculosis who were receiving anti-tuberculosis therapy (ATT) for at least one month were eligible. Patients were excluded if they had pre-existing kidney disease; diabetes mellitus, cardiovascular disease, or hypertension; a history of gout or hyperuricemia; use of medications known to affect serum uric acid or creatinine other than ATT; or pregnancy or lactation. Ninety-six consecutive eligible patients were enrolled, and all were included in the final analysis. Relevant demographic, diagnostic, and drug-resistance information was verified against available primary clinical, laboratory, and diagnostic records; discrepancies were resolved using the source records, without alteration of biochemical results.

Of the 96 participants, 54 (56.3%) had pulmonary tuberculosis and 42 (43.8%) had extrapulmonary tuberculosis (Table 2). Among the pulmonary cases, diagnosis was confirmed by GeneXpert MTB/RIF (*Mycobacterium tuberculosis*/rifampicin) in 49 and by sputum smear microscopy in five. Among the extrapulmonary cases, diagnosis was confirmed by tissue histopathology or cytology in 20 (lymph node biopsy, neck biopsy, pleural biopsy, omentum biopsy, or fine-needle aspiration cytology) and by site-appropriate imaging in three; for the remaining 19, the original diagnostic report was not retrievable from the study records, and tuberculosis had been diagnosed by the treating clinician on clinical and radiological grounds. Rifampicin-resistance testing was available for 50 participants; 35 had no resistance detected, and 15 had indeterminate results. No patient was diagnosed with rifampicin-resistant or multidrug-resistant tuberculosis, and none required second-line treatment during the study period.

Anti-tuberculosis treatment regimen

Participants were receiving first-line anti-tuberculosis therapy according to the treating team's clinical records. Detailed drug charts were not independently verified as part of this study; therefore, individual pyrazinamide exposure, adherence, and exact regimen timing could not be confirmed. For the exploratory comparison, recorded treatment durations of ≤3 months and >3 months were used as operational grouping variables. These duration-defined groups should not be interpreted as verified intensive- and continuation-phase regimens.

Specimen collection and processing

Approximately 5 mL of venous blood was collected in serum separator tubes using standard precautions. Samples were transported to the laboratory within approximately 10 minutes and were allowed to clot at room temperature. Serum was separated by centrifugation at 3000-4000 rpm for five minutes. Clear, nonhemolyzed serum was transferred to appropriately labeled tubes. Samples with hemolysis or inadequate volume were not analyzed. Serum was analyzed promptly or stored at 2-8°C for up to 24 hours according to the study protocol.

Biochemical analysis and quality control

Serum uric acid was measured using an enzymatic uricase-peroxidase method. Serum creatinine was measured using the Jaffe kinetic alkaline picrate method. Both tests were performed on a Siemens Atellica automated chemistry analyzer according to the manufacturer's recommended procedures. Internal quality control was performed using normal and pathological control sera with each analytical batch, and calibration was performed when required. The laboratory reference intervals were 3.4-7.1 mg/dL for serum uric acid and 0.2-1.2 mg/dL for serum creatinine.

Statistical analysis

Continuous variables were summarized using mean ± standard deviation and median (interquartile range). Normality was assessed using the Shapiro-Wilk test [13], which indicated departure from normality for all continuous variables (age, treatment duration, serum uric acid, and serum creatinine; all p < 0.01). Nonparametric methods were therefore used throughout. Categorical variables were summarized as frequencies and percentages. Hyperuricemia was defined as serum uric acid >7.1 mg/dL and elevated creatinine as serum creatinine >1.2 mg/dL according to the laboratory reference intervals. Spearman rank correlation was used to assess the association between treatment duration and serum uric acid or creatinine. For the exploratory duration-group comparison, participants receiving treatment for ≤3 months were categorized as the ≤3-month group and those receiving treatment for >3 months as the >3-month group. The Mann-Whitney U test was used to compare continuous biochemical measures between groups, and Fisher's exact test was used to compare the frequency of hyperuricemia. The Mann-Whitney U test was also used to compare serum uric acid and creatinine by sex, tuberculosis category (new versus relapse), and tuberculosis site (pulmonary versus extrapulmonary). Treatment duration and both biochemical measurements were assessed at a single time point for each participant, so all analyses involving treatment duration compare different individuals at different stages of treatment rather than the same individuals over time. Because several exploratory comparisons were performed, no adjustment for multiple testing was applied, and the resulting p-values should be regarded as descriptive rather than confirmatory. Ninety-five percent confidence intervals for proportions were calculated using the Wilson score method and for correlation coefficients using the Fisher z transformation. A p-value < 0.05 was considered statistically significant. Statistical analyses were performed using IBM SPSS Statistics for Windows, Version 23 (Released 2015; IBM Corp., Armonk, New York).

Ethical considerations

The study was approved by the Institutional Ethics Committee of King Edward Medical University, Lahore (approval number 804/RC/KEMU). Written informed consent was obtained from all participants before sample collection. Patient confidentiality and anonymity were maintained throughout the study.

## Results

A total of 96 participants were included in the final analysis. The mean age was 30.15 ± 12.75 years (median 26.5, IQR 19.0-37.0), and the mean treatment duration was 4.38 ± 2.92 months (median 4.0, IQR 2.0-6.0). Fifty-four participants (56.3%) were female, and 42 (43.8%) were male. Fifty-four participants (56.3%) had pulmonary tuberculosis, and 42 (43.8%) had extrapulmonary tuberculosis. Seventy-seven participants (80.2%) were newly diagnosed TB cases, and 19 (19.8%) were relapse cases. Forty-five participants (46.9%) had received treatment for ≤3 months, and 51 (53.1%) had received treatment for >3 months. Participant characteristics and biochemical parameters are summarized in Table 1, and the distribution of sex, tuberculosis site, tuberculosis category, and treatment-duration group is presented in Table 2. 

**Table 1:** Participant Characteristics and Biochemical Parameters (n=96)

Variable	Mean ± SD	Median (IQR)
Age (years)	30.15 ± 12.75	26.5 (19.0–37.0)
Treatment duration (months)	4.38 ± 2.92	4.0 (2.0–6.0)
Serum uric acid (mg/dL)	5.62 ± 2.51	5.0 (3.9–6.625)
Serum creatinine (mg/dL)	0.71 ± 0.18	0.72 (0.6175–0.8025)

**Table 2:** Sex, Tuberculosis Site, Tuberculosis Category, and Treatment-Duration Group (n=96)

Variable	N	%
New TB cases	77	80.2
Relapse cases	19	19.8
≤3-month treatment group	45	46.9
>3-month treatment group	51	53.1
Female	54	56.3
Male	42	43.8
Pulmonary tuberculosis	54	56.3
Extrapulmonary tuberculosis	42	43.8

Serum uric acid and creatinine

The mean serum uric acid concentration was 5.62 ± 2.51 mg/dL, with a median of 5.00 mg/dL (IQR 3.90-6.63). Fourteen participants (14.6%) had concentrations below 3.4 mg/dL, 63 (65.6%) were within the laboratory reference interval, and 19 (19.8%; 95% CI 13.1-28.9%) had hyperuricemia (>7.1 mg/dL). The mean serum creatinine concentration was 0.71 ± 0.18 mg/dL, with a median of 0.72 mg/dL (IQR 0.62-0.80); ninety-four participants (97.9%) had creatinine values within the laboratory reference interval, and two (2.1%; 95% CI 0.6-7.3%) had elevated creatinine (>1.2 mg/dL). The distributions of serum uric acid and creatinine categories are shown in Table 3. 

**Table 3:** Distribution of Serum Uric Acid and Creatinine Categories (n = 96)

Category	N	%
Serum uric acid		
Low (<3.4 mg/dL)	14	14.6
Reference range (3.4–7.1 mg/dL)	63	65.6
High (>7.1 mg/dL)	19	19.8
Total	96	100.0
Serum creatinine		
Low (<0.2 mg/dL)	0	0.0
Reference range (0.2–1.2 mg/dL)	94	97.9
High (>1.2 mg/dL)	2	2.1
Total	96	100.0

Association between treatment duration and biochemical parameters

Treatment duration had a statistically significant inverse correlation with serum uric acid (Spearman ρ = −0.213, 95% CI −0.396 to −0.013, p = 0.037). In practical terms, participants with a longer recorded treatment duration tended to have lower serum uric acid concentrations. Treatment duration was not significantly correlated with serum creatinine (Spearman ρ = 0.066, 95% CI −0.136 to 0.263, p = 0.522). These associations are summarized in Table 4. 

**Table 4:** Association Between Treatment Duration and Biochemical Parameters

Outcome	Spearman ρ	p-value	Interpretation
Serum uric acid	-0.213	0.037	Significant inverse association
Serum creatinine	0.066	0.522	No significant association

Exploratory treatment-duration group analysis

Serum uric acid was higher among participants treated for ≤3 months than among those treated for >3 months (6.52 ± 3.14 vs. 4.82 ± 1.39 mg/dL; Mann-Whitney U test, p = 0.005). Hyperuricemia was found in 16 of 45 participants (35.6%; 95% CI 23.2-50.2%) in the ≤3-month group compared with 3 of 51 (5.9%; 95% CI 2.0-15.9%) in the >3-month group (Fisher's exact test, p < 0.001). Serum creatinine did not differ significantly between the two groups (0.71 ± 0.19 vs. 0.72 ± 0.17 mg/dL; Mann-Whitney U test, p = 0.670). These results are presented in Table 5. 

**Table 5:** Biochemical Parameters by Treatment-Duration Group Continuous variables (uric acid, creatinine) were compared between the ≤3-month and >3-month treatment-duration groups using the Mann–Whitney U test; the difference in the frequency of hyperuricemia was compared using Fisher's exact test. Data are presented as mean ± SD or n (%).

Parameter	≤3-month group (n=45)	>3-month group (n=51)	Test statistic	p value
Uric acid, mg/dL	6.52 ± 3.14	4.82 ± 1.39	U = 768.0	0.005
Creatinine, mg/dL	0.71 ± 0.19	0.72 ± 0.17	U = 1089.0	0.670
Hyperuricemia, n (%)	16 (35.6)	3 (5.9)		<0.001

Comparisons by sex, tuberculosis category, and tuberculosis site

Serum uric acid and creatinine were also compared by sex, tuberculosis category, and tuberculosis site. Male participants had higher serum uric acid than female participants (6.46 ± 2.86 vs. 4.96 ± 1.99 mg/dL; Mann-Whitney U test, p = 0.002) and higher serum creatinine (0.78 ± 0.15 vs. 0.66 ± 0.18 mg/dL; Mann-Whitney U test, p < 0.001). Serum uric acid did not differ significantly between new and relapse cases (5.44 ± 2.39 vs. 6.32 ± 2.90 mg/dL; p = 0.272), nor did serum creatinine (0.71 ± 0.19 vs. 0.75 ± 0.13 mg/dL; p = 0.211). Serum uric acid also did not differ significantly between participants with pulmonary and extrapulmonary tuberculosis (5.41 ± 2.67 vs. 5.89 ± 2.30 mg/dL; p = 0.076), nor did serum creatinine (0.71 ± 0.16 vs. 0.72 ± 0.20 mg/dL; p = 0.897). These comparisons are presented in Table 6. 

**Table 6:** Serum Uric Acid and Creatinine by Sex, Tuberculosis Category, and Tuberculosis Site (n = 96) Groups were compared using the Mann–Whitney U test. Data are presented as mean ± SD.

Comparison	Group 1, Mean ± SD	Group 2, Mean ± SD	Test statistic	p value
Uric acid - Male (n = 42) vs. Female (n = 54)	6.46 ± 2.86	4.96 ± 1.99	U = 1546.5	0.002
Creatinine - Male (n = 42) vs. Female (n = 54)	0.78 ± 0.15	0.66 ± 0.18	U = 1685.0	<0.001
Uric acid - New (n = 77) vs. Relapse (n = 19)	5.44 ± 2.39	6.32 ± 2.90	U = 611.5	0.272
Creatinine - New (n = 77) vs. Relapse (n = 19)	0.71 ± 0.19	0.75 ± 0.13	U = 595.0	0.211
Uric acid - Pulmonary (n = 54) vs. Extrapulmonary (n = 42)	5.41 ± 2.67	5.89 ± 2.30	U = 893.5	0.076
Creatinine - Pulmonary (n = 54) vs. Extrapulmonary (n = 42)	0.71 ± 0.16	0.72 ± 0.20	U = 1116.0	0.897

## Discussion

In this study of 96 patients receiving anti-tuberculosis treatment, the mean serum uric acid was 5.62 mg/dL, and the mean serum creatinine was 0.71 mg/dL. Hyperuricemia was found in 19.8% of the participants, while elevated creatinine was found in 2.1%. Recorded treatment duration showed a weak inverse association with serum uric acid, whereas no significant association was found with creatinine.

The frequency of hyperuricemia observed in this study is compatible with the known effect of pyrazinamide-containing treatment on uric acid metabolism. Pyrazinamide and its metabolites can reduce renal uric acid excretion [4,5]. Previous studies have reported increases in serum uric acid during the early period of pyrazinamide-containing treatment and decreases after pyrazinamide was withdrawn [6,8]. More recent data are consistent with this pattern: a prospective cohort study from Indonesia reported a significant rise in uric acid during early treatment [12], and a 2025 study from the same region found hyperuricemia in approximately half of patients receiving anti-tuberculosis therapy [14]. Elevated uric acid also has clinical relevance beyond tuberculosis treatment, having been linked to renal and cardiovascular risk in other populations [15]. However, in a large single-center cohort of tuberculosis patients, hyperuricemia during treatment was not independently associated with major adverse cardiovascular events [16]. Epidemiological data have also documented hyperuricemia as an adverse reaction to pyrazinamide-containing anti-tuberculosis therapy [17], while gout attacks have been reported as a clinical complication during anti-tuberculosis treatment [18]. In the present study, however, pyrazinamide exposure was not independently verified, so the observed frequency of hyperuricemia cannot be attributed specifically to pyrazinamide.

The duration-group comparison showed lower mean serum uric acid concentrations and a lower frequency of hyperuricemia among participants treated for more than three months than among those treated for three months or less (5.9% vs 35.6%; p < 0.001). Because this was a cross-sectional comparison of different individuals, the finding cannot determine whether uric acid concentrations declined after any particular drug was discontinued. The difference is compatible with the known pharmacological effect of pyrazinamide-containing treatment, but it does not establish that pyrazinamide exposure caused the higher uric acid concentrations in the ≤3-month group because individual drug exposure was not verified. Other differences between participants at different treatment durations may also contribute.

The inverse correlation between treatment duration and serum uric acid was statistically significant but weak (ρ = −0.213). Because this correlation is based on a cross-sectional comparison across participants with different treatment durations rather than repeated measurements within the same patients, it should not be interpreted as evidence of a causal or temporal effect of treatment duration on serum uric acid but rather as an association observed across the study population. This also means that treatment duration alone is unlikely to explain most of the variation in serum uric acid. Other factors, such as age, sex, diet, hydration, underlying illness, adherence to treatment, and concomitant medicines, may also affect uric acid levels. These factors were not assessed in detail in the present dataset.

Exploratory comparisons across other patient characteristics yielded mixed results. Male participants had significantly higher serum uric acid and serum creatinine than female participants. Because higher baseline serum uric acid and creatinine levels in men than in women, largely attributed to estrogen-related enhancement of renal urate clearance and greater muscle mass, are well established in the general population independent of tuberculosis or its treatment [19], this difference may simply reflect underlying sex-based physiology rather than a treatment-specific effect, and the present cross-sectional design cannot distinguish between the two explanations. No significant difference in serum uric acid or creatinine was found between new and relapse cases or between participants with pulmonary and extrapulmonary tuberculosis, suggesting that these characteristics were not major determinants of biochemical abnormality in this cohort.

Elevated creatinine was uncommon, with only two participants having values above the laboratory reference limit. This finding is generally consistent with reports in which creatinine remained relatively stable during anti-tuberculosis treatment despite increases in serum uric acid [8]. However, the small number of participants with elevated creatinine does not allow conclusions about predictors of renal abnormalities. In addition, serum creatinine alone is not sufficient to diagnose acute kidney injury [9].

One strength of this study was the use of patient-level laboratory data from 96 participants in a tertiary-care hospital. Standardized biochemical methods, defined laboratory reference intervals, and routine internal quality-control procedures were used. This dataset also allowed the association between treatment duration and the biochemical parameters to be assessed statistically. Recent reviews of adverse reactions to anti-tuberculosis therapy have also emphasized that clinically important toxicity is common during treatment and that monitoring may be relevant during periods of greater toxicity risk [20-22]. Contemporary kidney disease guidance further emphasizes that acute kidney injury requires a change in creatinine or urine output over time rather than an isolated elevated creatinine value [23].

Limitations

This study has several limitations. Its cross-sectional design and absence of pretreatment values preclude assessment of within-patient changes or causal treatment effects. Consecutive nonprobability sampling from a single tertiary-care hospital may limit generalizability. Treatment phase, adherence, and individual drug exposure were not verified; therefore, the ≤3-month and >3-month groups represent duration-defined rather than confirmed treatment-phase groups. Rifampicin-resistance testing was incomplete, so undetected resistance cannot be fully excluded. Exploratory subgroup analyses were unadjusted for multiple testing, and the small number of participants with elevated creatinine limited assessment of its predictors. Potential confounders such as BMI, diet, hydration, and concomitant medications were not recorded.

## Conclusions

This study provides local, patient-level data on biochemical parameters during first-line anti-tuberculosis therapy at a tertiary-care hospital in Lahore, Pakistan, in a cohort that included both pulmonary and extrapulmonary tuberculosis. Mean serum uric acid (5.62 mg/dL) and mean serum creatinine (0.71 mg/dL) both fell within the laboratory reference ranges. Hyperuricemia was present in 19.8% of participants and was more frequent among those treated for ≤3 months than among those treated for >3 months (35.6% vs. 5.9%), while elevated creatinine was uncommon at 2.1%. Because treatment regimen and pyrazinamide exposure were not independently verified and the two duration-defined groups comprised different individuals assessed at a single time point, this difference should not be interpreted as evidence of a treatment-caused or phase-specific increase in uric acid. The absence of pretreatment measurements also means that pre-existing hyperuricemia and selection differences between groups cannot be excluded. These findings provide local data on biochemical abnormalities during routine first-line therapy; prospective studies with serial measurements and verified drug exposure are needed to assess treatment-related changes.
